# UWB Indoor Localization Based on Artificial Rabbit Optimization Algorithm and BP Neural Network

**DOI:** 10.3390/biomimetics10060367

**Published:** 2025-06-04

**Authors:** Chaochuan Jia, Can Tao, Ting Yang, Maosheng Fu, Xiancun Zhou, Zhendong Huang

**Affiliations:** 1 College of Electronics and Information Engineering, West Anhui University, Lu’an 237000, China; 03000076@wxc.edu.cn (C.J.); fums@wxc.edu.cn (M.F.); zhouxc@wxc.edu.cn (X.Z.); 2 Anhui Province Intelligent Hydraulic Machinery Joint Construction Subject Key Laboratory, Lu’an 237000, China; hzd3276032@163.com; 3 College of Electrical and Optoelectronic Engineering, West Anhui University, Lu’an 237000, China; 04000075@wxc.edu.cn

**Keywords:** artificial rabbit optimization algorithm, BP neural network, non-line-of-sight, UWB, line-of-sight

## Abstract

In the field of ultra-wideband (UWB) indoor localization, traditional backpropagation neural networks (BPNNs) are limited by their susceptibility to local minima, which restricts their ability to achieve global optimization. To overcome this challenge, this paper proposes a novel hybrid algorithm, termed ARO-BP, which integrates the Artificial Rabbit Optimization (ARO) algorithm with a BPNN. The ARO algorithm optimizes the initial weights and thresholds of the BPNN, enabling the model to escape local optima and converge to a global solution. Experiments were conducted in both line-of-sight (LOS) and non-line-of-sight (NLOS) environments using a four-base-station configuration. The results demonstrate that the ARO-BP algorithm significantly outperforms traditional BPNNs. In LOS conditions, the ARO-BP model achieves a localization error of 6.29 cm, representing a 49.48% reduction compared to the 12.45 cm error of the standard BPNN. In NLOS scenarios, the error is further reduced to 9.86 cm (a 46.96% improvement over the 18.59 cm error of the baseline model). Additionally, in dynamic motion scenarios, the trajectory predicted by ARO-BP closely aligns with the ground truth, demonstrating superior stability. These findings validate the robustness and precision of the proposed algorithm, highlighting its potential for real-world applications in complex indoor environments.

## 1. Introduction

Recent advancements in science and technology have propelled location-based services (LBSs) to have a pivotal role in modern society, driving commercialization and widespread adoption across diverse domains. Applications such as map navigation, forest fire prevention, urban construction, and traffic management critically depend on LBS, enhancing societal productivity and individual well-being [1]. In outdoor environments, Global Navigation Satellite Systems (GNSSs), including the Global Positioning System (GPS) and BeiDou Navigation Satellite System (BDS), deliver meter-level positioning accuracy, enabling ubiquitous applications ranging from navigation to logistics [2]. However, these systems face significant limitations in indoor environments due to signal attenuation and multipath interference. Notably, individuals spend 70–90% of their time indoors [3], where the demand for reliable LBSs—such as indoor navigation, asset tracking, and emergency response—remains unmet [4]. The inability of conventional GNSSs to provide precise indoor localization has emerged as a critical barrier to realizing seamless location-aware services. To overcome this challenge, both academia and industry are intensively investigating high-precision indoor positioning technologies capable of addressing complex electromagnetic disturbances and spatial constraints, thereby bridging the gap between outdoor and indoor positioning capabilities [5].

In parallel with global advancements in indoor positioning research conducted by international institutions [6], China has strategically launched multiple national key research and development (R&D) programs under the broader framework of Earth observation initiatives, specifically targeting indoor positioning challenges [7]. With substantial support from the National Key R&D Program, a consortium comprising Chinese industrial enterprises, academic institutions, and research organizations has collaboratively conducted systematic investigations into indoor positioning technologies [8]. This coordinated national effort focuses on two primary objectives: (1) advancing fundamental technologies for indoor spatial awareness and (2) optimizing service performance metrics, particularly with regard to positioning accuracy (sub-meter level) and operational reliability under complex architectural conditions [9].

The field of indoor positioning technology has witnessed substantial methodological diversification, with current research spanning eleven principal technical approaches [10]: base station localization, Wi-Fi fingerprinting [11], radio frequency identification (RFID) systems, Bluetooth low-energy (BLE) beacons, ultra-wideband (UWB) time-of-flight measurements, ZigBee mesh networks, geomagnetic anomaly mapping, digital terrestrial broadcast signal processing, pseudo-satellite (indoor GPS analog) systems, computer vision-based spatial recognition, and visible light communication (VLC) modulation [12]. These heterogeneous approaches leverage distinct signal modalities and protocol frameworks [13], including but not limited to cellular base station triangulation [14], Wi-Fi-received signal strength indicators (RSSIs), RFID tag–reader interactions, Bluetooth channel state information (CSI), UWB pulse characterization, ZigBee protocol stacks, geomagnetic field variations, digital signal correlation techniques, simulated satellite signal transmission, visual odometry algorithms, and optical intensity modulation schemes [15]. This technological plurality addresses diverse operational requirements across application domains (e.g., smart infrastructure, industrial automation, and emergency response systems), thereby establishing an evolutionary trajectory marked by progressive accuracy enhancement (sub-decimeter resolution in UWB systems) and adaptive multi-sensor fusion architectures [16].

The diversity of indoor positioning techniques offers tailored solutions for various scenarios [17]. Base station and Wi-Fi positioning are commonly used in indoor applications [18], but their accuracy is relatively low due to signal occlusion and multipath effects. RFID technology is suitable for the precise positioning of specific objects [19], but it requires a large number of RFID tags, which can be costly. Bluetooth positioning is an ideal choice for mobile device localization due to its low power consumption and relatively high precision [20], though it necessitates the deployment of numerous Bluetooth devices indoors. ZigBee, with its low power consumption and cost, is suitable for low data rate communication and positioning [21], but its accuracy is limited, and its applicability is restricted. Geomagnetic positioning is unaffected by illumination but depends on stable magnetic fields [22], making it susceptible to environmental disturbances. Pseudo-satellite localization performs well in scenarios requiring sub-meter precision [23], but it demands additional hardware support and incurs higher costs. Computer vision and visible light localization are applicable to various scenarios; however, the former requires a large number of cameras and complex algorithms, while the latter is constrained by lighting conditions [24,25]. Thus, selecting the appropriate indoor positioning technology requires a comprehensive consideration of factors such as application scenarios, accuracy requirements, cost constraints, and equipment limitations to meet specific needs [26]. Among the many indoor positioning technologies, ultra-wideband (UWB) positioning is considered one of the most promising solutions due to its numerous advantages [27], including high bandwidth, high resolution, high transmission rate, strong robustness, low power consumption, and relatively low cost [28]. The mainstream UWB positioning algorithms include time-of-flight (TOF)- [29], Time Difference of Arrival (TDOA)- [30], Angle of Arrival (AOA)- [31], and received signal strength (RSS)-based positioning algorithms [32].

The UWB positioning system is anticipated to achieve centimeter-level precision. However, the complexity of indoor environments significantly impacts its accuracy due to non-line-of-sight errors such as multipath effects. Scholars worldwide have conducted various studies to enhance UWB positioning accuracy [33]. For instance, some studies have employed the least-squares method to identify and eliminate observations with large residuals or gross errors [34], though this method does not effectively address systematic errors. Another approach involves using a Kalman filter algorithm to analyze the characteristics of UWB non-line-of-sight errors [35]. This algorithm identifies errors using a sliding window and dynamically adjusts noise parameters for error compensation. However, these methods alone are insufficient and often require additional techniques, such as function fitting, to correct systematic errors. Additionally, integrating UWB with inertial sensors has been explored as a means to mitigate the impact of non-line-of-sight errors [36]. With the continuous advancement of artificial intelligence in both theory and technology, its application scope has expanded. Scholars have proposed establishing neural network models for the nonlinear calibration of RF signals. These neural network calibration models can be developed through repeated training. However, this approach necessitates setting multiple parameters, which is currently carried out through experience-based selection. If the parameters are not carefully chosen, the calibration effect may be suboptimal [37,38].

Existing UWB error correction methods have several limitations, including a narrow application scope, limited correction accuracy, and complex models [39]. This paper introduces a novel hybrid algorithm called ARO-BP, which combines the Artificial Rabbit Optimization (ARO) algorithm with the backpropagation (BP) neural network. By using the ARO to generate random weights and thresholds for the BP neural network, the issue of the BP algorithm becoming trapped in local optima can be effectively mitigated. To evaluate the performance of the ARO-BP algorithm across different environments, we conducted experiments in various scenarios, including LOS (line-of-sight), NLOS (non-line-of-sight), and autonomous vehicle motion. The superiority of the ARO-BP algorithm is demonstrated through a comparison of its localization accuracy with that of the traditional BP neural network in a UWB system.

The primary contribution of this paper is the introduction of a novel hybrid algorithm model, ARO-BP, specifically designed to optimize UWB indoor positioning. This contribution includes three significant innovations:

(1) The development of an innovative hybrid localization algorithm model, ARO-BP.
(2) The integration of the BP neural network with the ARO algorithm to create a new method for optimizing the neural network structure, which enhances the accuracy and anti-jamming capability of the UWB indoor positioning system.
(3) The substantial reduction in the original positioning error in the UWB positioning system, as demonstrated through the application and validation of the algorithm model in practical scenarios.

The structure of this paper is organized as follows: The second section introduces the principles of ultra-wideband (UWB) positioning, the BP neural network, and the Artificial Rabbit Optimization (ARO) algorithm and explains how the ARO-BP model is constructed by combining ARO with the BP neural network. The third section presents detailed experimental results in LOS, NLOS, and autonomous vehicle environments. Finally, the fourth section summarizes the research findings.

## 2. Model Fundamentals of Positioning

### 2.1. The UWB Localization Principle

**Figure 1:**
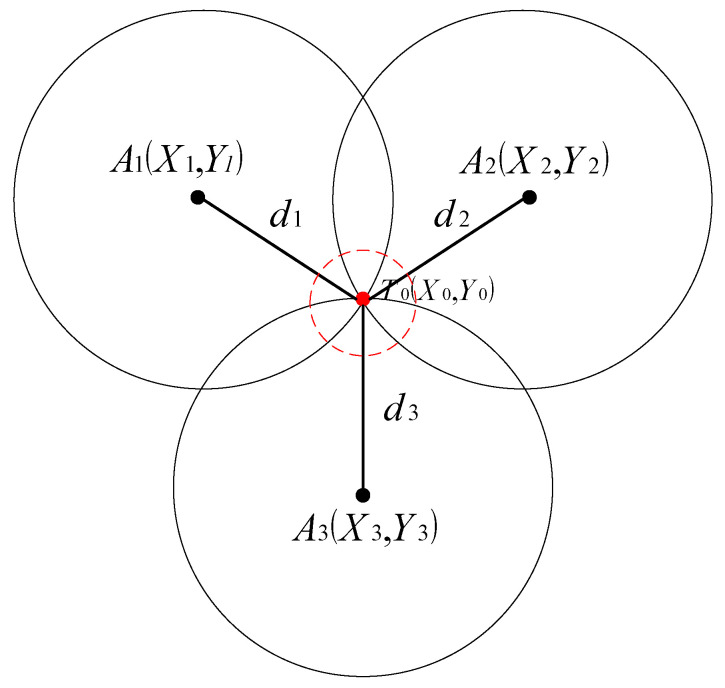
Principle of the three-sided positioning algorithm.

The multilateral positioning algorithm is a method used for determining the location of a target based on distance measurements from the target to three or more known reference points [40]. These reference points, also known as anchor points or base stations, are used to calculate the target’s position. To simplify the explanation, let us consider a scenario where three base stations, labeled $A_{1}\left(x_{1},y_{1}\right)$, $A_{2}\left(x_{2},y_{2}\right),$ and $A_{3}\left(x_{3},y_{3}\right)$, are located at known positions. The distances between the target $T_{0}$($x_{0},y_{0}$) and each base station, measured using UWB, are represented as $d_{1}$, $d_{2}\;and\;d_{3}$, respectively, as shown in Figure 1.

In the two-dimensional experimental scenario, using the Pythagorean theorem, the formula for the position of the label $T_{n}$ can be obtained.

Under ideal conditions, the position of the label $T_{n}$ corresponds to the unique exact solution, which is shown as a red dot in Figure 1.

In practical scenarios, there can be errors in the measurement of the distance value d and the coordinates of base station A. These errors can occur due to various factors such as the measurement instrument itself, signal propagation attenuation, and the presence of indoor or complex environments. In such cases, the signal may experience multiple paths, resulting in errors in the measurement results [41]. Consequently, the coordinates of the solved label $T_{n}$ are not confined to a single point but represent a range of possible positions. Therefore, it is necessary to find the best solution within this area, as shown by the red circle in Figure 1.

### 2.2. BP Neural Network

**Figure 2:**
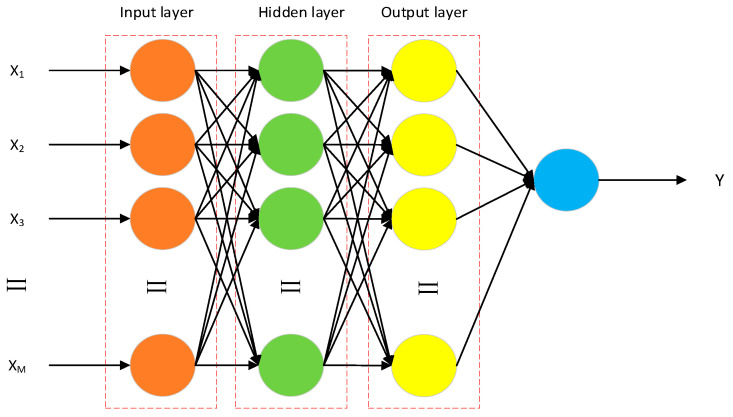
Topology of BP neural network.

The backpropagation neural network (BPNN) is a widely utilized artificial neural network model in the fields of machine learning and deep learning [42]. It comprises a fixed input layer, several adjustable hidden layers, and an output layer. The number of nodes within the hidden layers can be flexibly configured to match the complexity of the problem at hand [43]. In this study, we consider a simplified three-layer network model, as depicted in Figure 2. A key feature of the BPNN is its signal propagation mechanism, which includes forward signal propagation and error backpropagation. If the output does not meet the desired objective, the network initiates the backpropagation algorithm to perform error correction and parameter tuning [44]. This process involves propagating the error signal backward through the network and adjusting the model by modifying the weights between the layers. By comparing the actual output with the target output, the network optimizes its performance over time through iterative adjustments of the connection weights. This training process continues until the network’s output satisfies the expected requirements or reaches a predefined maximum number of iterations. In neural networks, the fundamental functional unit is the neuron, and the interconnection of multiple neurons forms the entire network structure [45]. This architecture enables the network to process and learn complex input–output relationships effectively.

### 2.3. Model Building

Based on the localization principle above, localization using a BP neural network alone leads to a high risk of falling into local optimal solutions due to the problem of the initial weights and thresholds of the BP neural network. Therefore, in this section, we propose to use the ARO algorithm to optimize the weights and thresholds of the BP neural network. The ARO algorithm is a new bio-inspired meta-heuristic algorithm proposed in 2022. The algorithm combines the meandering foraging and stochastic hiding strategies of rabbits, as well as variations in energy levels, to develop a new optimizer. It shows significant advantages in solving benchmark functions and engineering problems, and ARO outperforms other algorithms in optimizing the work of BP networks using the feature vectors of the training sample set [46].

The parameters related to the ARO algorithm used in this paper are as follows: population size—50; energy coefficient—α = 0.8; maximum number of iterations—100; and energy decay rate—β = 0.95.

The ARO-BP algorithm represents a novel approach that integrates the Artificial Rabbit Optimization (ARO) algorithm with a backpropagation neural network (BPNN). This algorithm combines the extensive and targeted exploration capabilities of ARO with the nonlinear fitting and approximation strengths of a BPNN, enhancing both optimization performance and prediction accuracy [47]. The ARO-BP algorithm merges the search strategy of the ARO algorithm with the training and optimization functions of the BP neural network, following these steps:

1. Rabbit Position and Energy Factor Initialization: the initial position of the rabbit is determined using the ARO algorithm’s initialization method, while the energy factor is set based on the specific characteristics of the problem.
2. Input Data Determination: the rabbit’s position serves as the input data for training and prediction within the BP neural network.
3. BP Neural Network Training: The input, derived from the rabbit’s position, is processed by the network. The difference between the predicted and actual output is used as the loss function, guiding the adjustment of network weights and biases through backpropagation to minimize errors.
4. Rabbit Position and Energy Factor Update: the position and energy factors of the rabbits are updated according to the ARO algorithm’s strategy, incorporating the outcomes of the BP network’s training.
5. Iterative Execution: steps 2 through 4 are repeated until the termination condition is met, either when the maximum number of iterations is reached or when the error falls below a predefined threshold.

By combining the global search abilities of ARO with the precision and accuracy of BP neural network training, the ARO-BP algorithm provides superior optimization and predictive performance for complex problems [48]. The application of the ARO-BP hybrid algorithm in UWB localization significantly enhances positioning accuracy and stability. The advantages and disadvantages of the ARO-BP algorithm and BP neural network algorithm are shown in Table 1.

**Figure 3:**
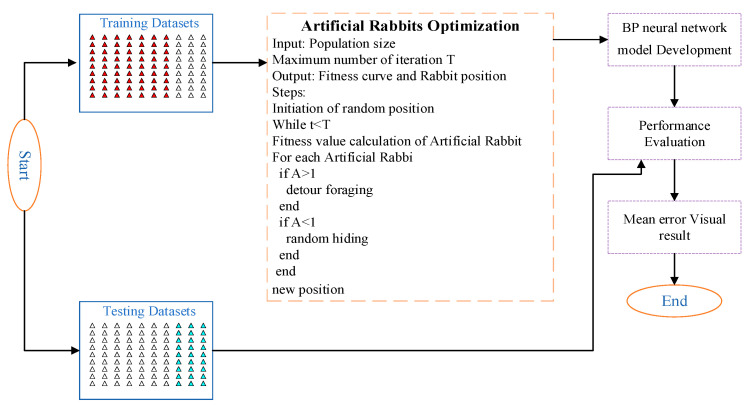
ARO-BP model of hybrid algorithm.

Figure 3 illustrates the evolution of the ARO-BP model. Initially, the retrieved database was divided into two distinct datasets: one for training and the other for validation. The training dataset was utilized to train the BPNN, while the validation dataset was employed to evaluate the model’s performance and accuracy. Random values were initially assigned to the BPNN’s weights and thresholds, and the training data were then fed into the network for training. Through backpropagation, the neural network adjusted the weight coefficients and decision boundaries based on the input data and corresponding target outputs, aiming to minimize the discrepancy between the predicted and actual results. Furthermore, the ARO algorithm was introduced to enhance the performance of the BP neural network. The ARO algorithm applied a global optimization strategy, combining stochastic and local search methods to refine the model. Ultimately, this process yielded an ARO-optimized BP neural network model. The test dataset was then input into this final model to generate predictions. To assess the model’s accuracy and reliability, the predicted results were compared with the actual coordinates, and the prediction error was calculated. This comprehensive process ensured that the ARO-BP model was effectively trained, optimized, and validated, making it well suited for predicting and optimizing localization data. The hybrid model, which integrates the ARO algorithm with the BP neural network, demonstrates exceptional performance and broad applicability.

Computational complexity is a pivotal measure for gauging the effectiveness of optimization algorithms, as it provides an estimate of the resources an algorithm will demand when confronting problems of differing scales. In the case of the Artificial Rabbit Optimization (ARO) algorithm, its efficiency is contingent upon several primary components: the number of agents (n), the degree of variable dimensionality in the problems (d), and the upper limit of the number of iterations (T). The comprehensive computational complexity of ARO can be articulated as indicated, $O\left(Tnd+Tn+n\right)$.

## 3. Performance Evaluation and Analysis

In order to verify the accuracy of UWB positioning and the effectiveness of the algorithms and to provide the necessary data for future research and development, we have set up experimental environments and conducted systematic experiments to evaluate their performance and practical feasibility. The UWB device used in this study operates at 3.2 GHz–6.9 GHz. In order to simulate various signal transmission conditions and realistically reproduce real-world application environments, we have carefully designed three specific test environments: the LOS scenario, NLOS scenario, and motion scenario.

### 3.1. LOS Environment Experiment

#### 3.1.1. Scene Configuration

To simulate an indoor positioning scenario, we installed four UWB base stations in a designated area. Each base station is positioned at a height of 2.2 m and located at distinct coordinates: (0,0), (0,5), (5,0), and (5,5). These base stations are used to provide known location information for indoor positioning.

**Figure 4:**
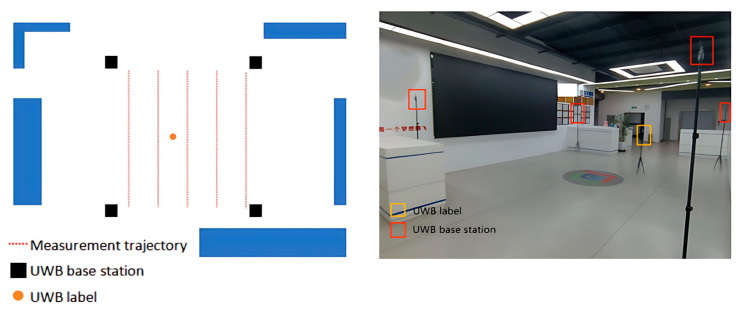
Schematic diagram of the LOS environment.

Within this environment, the UWB base station offers a stable reference frame for determining the location of the target object. By collaborating with upper-level computer software, we can capture the real-time location of UWB tags, enabling the recording and analysis of indoor positioning data. Figure 4 illustrates the experimental scenario.

#### 3.1.2. Analysis of Results

In a line-of-sight (LOS) environment, we conducted a series of experiments to evaluate the effectiveness of UWB positioning technology under varying altitude conditions. The experiments were performed at two distinct heights (1.0 m and 1.8 m) to ensure methodological rigor and experimental validity. The actual and measured positions were randomized to create sample sets, which were then divided into training and validation subsets. In this data division, the total number of samples was 96. Among them, 60 samples were selected as the training set, while the remaining 36 samples were used as the test set. Such a division ensures that the training set has enough data volume for model learning and also reserves a reasonable number of samples for the test set to evaluate the generalization ability of the model.

**Figure 5:**
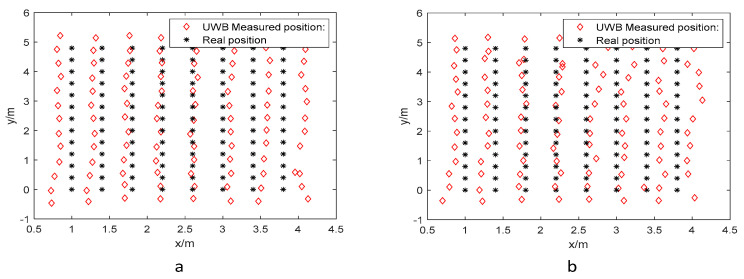
Actual coordinate position and UWB measurement data in the LOS environment: (**a**) 1.8 m and (**b**) 1.0 m.

Figure 5 illustrates the relationship between measured and actual coordinates for two UWB tag sets at different elevations, where black asterisks denote ground-truth positions and red diamonds represent measurements obtained from the host software. The limited overlap between these markers demonstrates significant positioning errors in conventional UWB techniques, particularly under specific spatial configurations.

**Figure 6:**
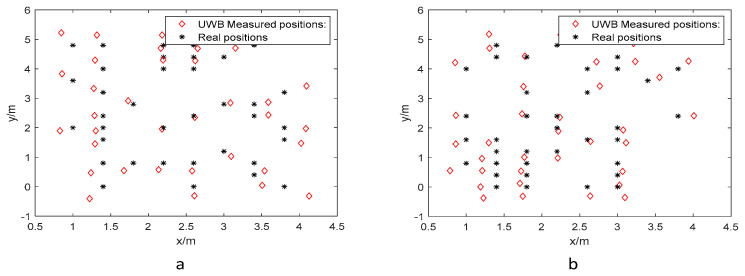
Training dataset in the LOS environment: (**a**) 1.8 m and (**b**) 1.0 m.

The training dataset (Figure 6) contains black dots, indicating actual coordinates and red dots showing UWB measurements. During training, we implemented a backpropagation algorithm to minimize prediction errors through weight and bias adjustments. Momentum terms and regularization techniques were incorporated to accelerate convergence and prevent model overfitting.

**Figure 7:**
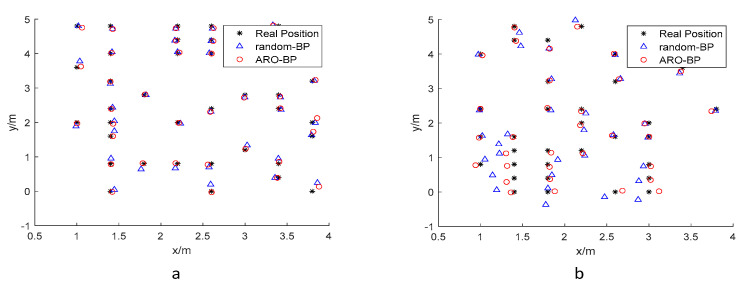
Test dataset in the LOS environment: (**a**) 1.8 m and (**b**) 1.0 m.

For model evaluation, randomly selected test points were processed through both BPNN and ARO-BP architectures to generate predicted positions. Figure 7 compares the actual coordinates (black asterisks) with predictions from the baseline BPNN (blue triangles) and optimized ARO-BP (red circles), with all coordinates expressed in meters.

**Figure 8:**
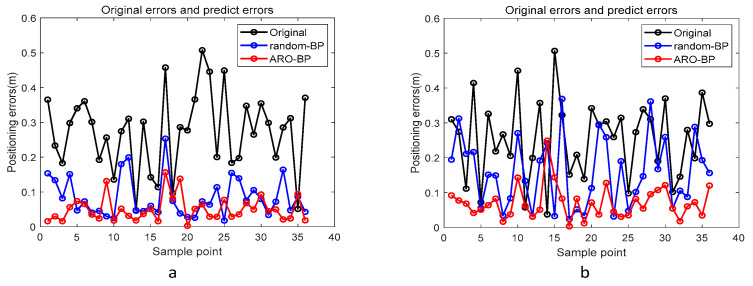
Error curves between predictable and actual values in the LOS environment: (**a**) 1.8 m and (**b**) 1.0 m.

**Figure 9:**
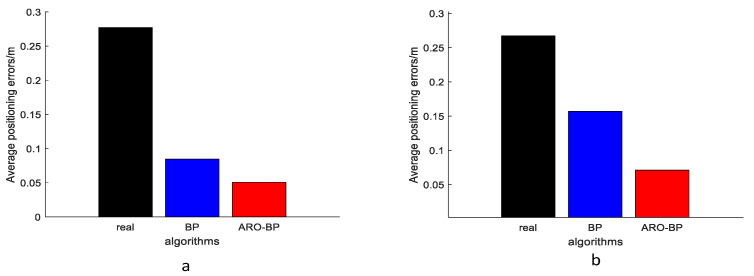
Histogram of the mean error of the test set in the LOS environment: (**a**) 1.8 m and (**b**) 1.0 m.

Performance metrics were analyzed through error distribution plots (Figure 8) and error progression curves (Figure 9) for both elevation levels. The results indicate that while the standard BPNN model improves upon raw UWB measurements (black data points), it frequently converges to suboptimal local solutions, as evidenced by residual prediction errors (blue markers). Conversely, the ARO-BP enhancement (red markers) demonstrates superior accuracy and stability, achieving consistent error reduction across test scenarios in LOS conditions.

From the experimental results, the average error of the data used for testing in the LOS environment at a height of 1.8 m is 27.71 cm, the average error of the BP model is 9.98 cm, and the average error of the ARO-BP model is 5.11 cm, which is obviously better than the BP model; specifically, the accuracy of the ARO-BP model is 17.58% higher than that of the traditional BP model at a 1 m height. The average error of the data used for the test is 25.47 cm. The average error of the BP model is 14.91 cm, while the average error of the ARO-BP model is 7.48 cm, which is obviously better than the BP model, and the accuracy of the ARO-BP model is 29.17% higher than that of the traditional BP model.

### 3.2. NLOS Environment Experiment

#### 3.2.1. Scene Configuration

**Figure 10:**
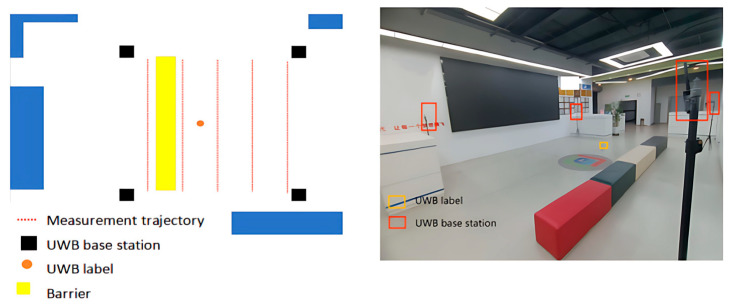
Schematic diagram of the NLOS environment.

In order to simulate a more challenging test environment, we reduced the mounting height of the four UWB base stations indoors from 2.2 m to 1.4 m and used the occlusion of objects such as sofa benches (5 × 0.5 m) to create localization test points. The four UWB base stations were placed indoors at the same locations, (0,0), (0,5), (5,0) and (5,5), with their heights uniformly adjusted to 1.4 m. This arrangement makes the signal propagation path more complex, potentially leading to interference and reflection from obstacles. The specific arrangement is shown in Figure 10.

#### 3.2.2. Analysis of Results

To realistically simulate the non-line-of-sight (NLOS) environment, we adjusted the height of four UWB base stations to 1.4 m and introduced interference objects such as sofa stools between the test points. We measured the localization coordinates of UWB tags at different heights (0.15 m and 0.45 m). These measured positions were compared with the actual positions to form a sample set. The data were then randomly mixed up and divided into separate training and testing datasets. In the NLOS environment, the total number of samples was still 96. Again, 60 samples were selected as the training set, and the remaining 36 samples were used as the test set.

**Figure 11:**
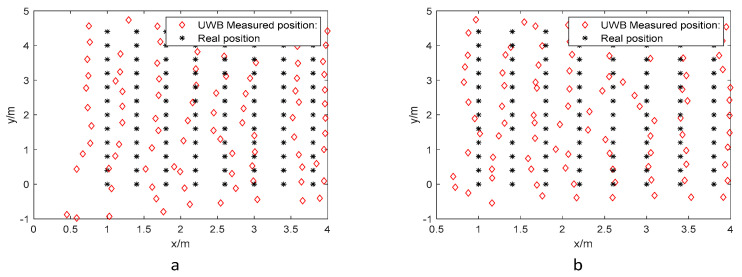
Actual coordinate positions and UWB measurements in NLOS environment: (**a**) 0.45 m and (**b**) 0.15 m.

Figure 11 shows the measured and actual coordinate positions of UWB tags at various heights in the NLOS environment. There is a noticeably low overlap between the black asterisks (actual positions) and the red diamonds (UWB positioning measurements). This discrepancy is primarily due to the multipath effect in the NLOS environment, which complicates signal propagation and degrades the performance of the localization algorithm. The multipath effect introduces randomness in the received signals, making it challenging to accurately estimate signal arrival time and resulting in increased localization errors. Consequently, optimizing the algorithm in such conditions is more complex and requires enhanced performance.

**Figure 12:**
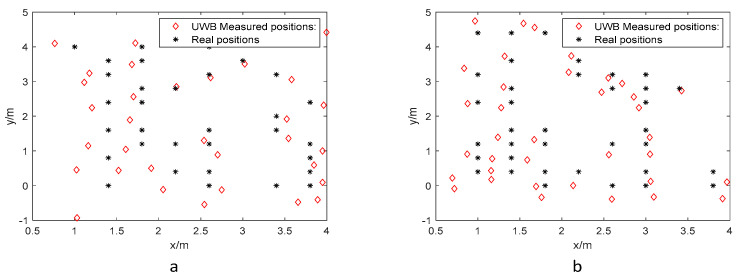
Training dataset in the NLOS environment: (**a**) 0.45 m and (**b**) 0.15 m.

**Figure 13:**
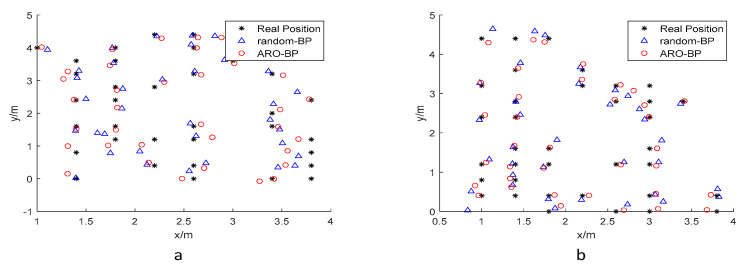
Test dataset in the NLOS environment: (**a**) 0.45 m and (**b**) 0.15 m.

Following the same methodology as in the LOS environment, we constructed a training dataset and a test dataset from the sample set. The training dataset was used to train both the BP neural network and ARO-BP models. Subsequently, the test dataset was input into the trained models to predict localization positions. The training and test datasets for the NLOS environment are shown in Figure 12 and Figure 13.

**Figure 14:**
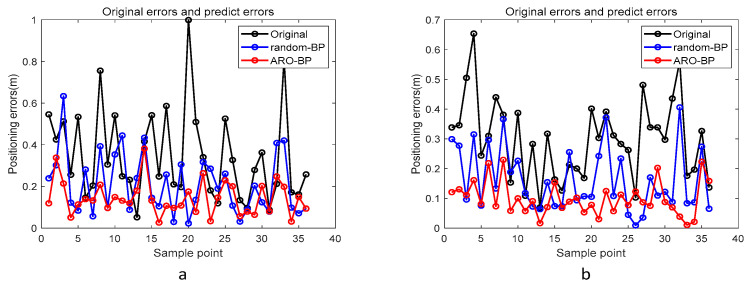
Error curves between predictable and actual values in the NLOS environment: (**a**) 0.45 m and (**b**) 0.15 m.

**Figure 15:**
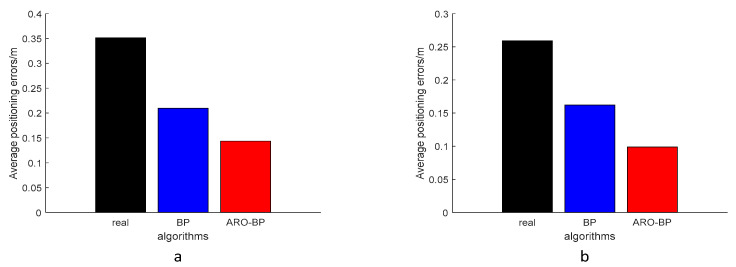
Histogram of the mean error of the test set in the NLOS environment: (**a**) 0.45 m and (**b**) 0.15 m.

Figure 14 presents the error curve between the predicted and actual values in the NLOS environment, while Figure 15 displays the average error bar graph of the test set in the NLOS environment. The empirical findings indicate that, under various height conditions in the NLOS environment, the ARO-BP model consistently demonstrates superior localization performance with notably lower error margins compared to the conventional BP neural network model.

From the experimental results, the average error of the data used for testing in the NLOS environment at a height of 0.45 m is 35.91 cm. The average error of the BP model is 20.81 cm, and that of the ARO-BP model is 12.78 cm, which is obviously better than the BP model, and the accuracy of the newly proposed model is 22.45% higher than that of the traditional BP model. The average error of the data used for the test is 25.93 cm at a height of 0.15 m. The average error of the BP model is 16.38 cm, while the average error of the ARO-BP model is 9.01 cm, which is obviously better than the BP model, and the accuracy of the ARO-BP model is 28.43% higher than that of traditional BP model.

### 3.3. Actual Motion Scene Experiment

#### 3.3.1. Scene Configuration

**Figure 16:**
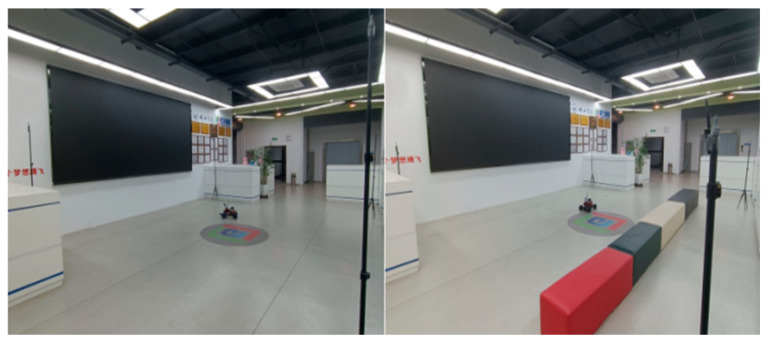
Motion scenes.

Through experiments conducted in LOS and NLOS environments, we have confirmed the advantages of the ARO-BP model in terms of localization accuracy and stability. To further assess the performance of the ARO-BP model in real-world motion scenarios, we integrated UWB tags into the unmanned vehicle, as illustrated in Figure 16.

The vehicle then executed circular motion and S-curve driving along the planned route in LOS and NLOS environments, respectively. The running trajectory of the vehicle was obtained using the BP neural network model and the ARO-BP model and compared with the real trajectory and the trajectory recorded by the UWB. This comparison is valuable in investigating whether the ARO-BP model can achieve high-precision localization in motion scenarios, assess the stability of localization, and identify any abnormal situation anomalies.

#### 3.3.2. Analysis of Results

**Figure 17:**
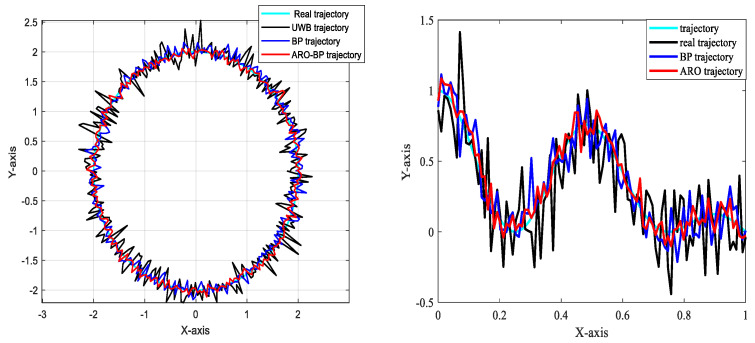
Motion trajectory map in LOS environment.

**Figure 18:**
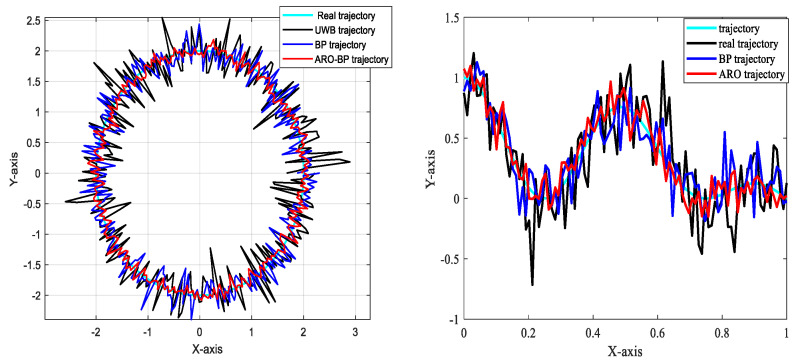
Motion trajectory map in NLOS environment.

Figure 17 and Figure 18 illustrate the motion trajectory maps of the driverless vehicle in indoor line-of-sight (LOS) and non-line-of-sight (NLOS) environments, respectively. These motion scenarios align with the experimental setups depicted in Figure 4 and Figure 10. In the experiments, the vehicle followed a circular path and traveled along an S-curve. The results indicate that the motion trajectory errors in the NLOS environment are significantly larger than those in the LOS environment.

In the NLOS environment, both the conventional BP neural network model and the enhanced ARO-BP model experience a decline in localization precision. However, the ARO-BP algorithm measures the motion trajectory more accurately and outperforms the BP neural network alone.

To summarize, the introduction of the ARO-BP model successfully addressed the fluctuation and bias issues inherent to traditional UWB localization techniques, resulting in significantly improved localization accuracy and system stability. This research supports the future development and widespread application of UWB localization technology. In real-world motion scenarios, the ARO-BP model demonstrates considerable potential and value.

## 4. Conclusions

In this paper, we present a novel indoor positioning algorithm designed for use in UWB localization systems, which combines the artificial rabbit algorithm (ARO) and BP neural network to form an ARO-BP intelligent optimization algorithm model. The objective is to enhance the precision of UWB indoor localization. The study first collected actual position coordinates and UWB measurement coordinates to obtain error data. Then, a BP neural network was trained to generate corresponding weights and thresholds. Following this, the ARO approach was adopted to maximize efficiency by optimizing the weights and thresholds in the BP neural network procedure until the error associated with localization reached its minimal value. Finally, a new ARO-BP algorithm model was established to calibrate the UWB measurement coordinates. This study conducted a comparison of the error performances between the conventional BP neural network and the ARO-BP algorithmic model.

The experimental results show that the BP neural network model suffers from the problem of falling into local optimal solutions when optimizing UWB indoor localization, while the ARO-BP algorithm model demonstrates higher accuracy and stability. Under different experimental environments and altitudes, the ARO-BP algorithm model exhibits a high optimization rate. In the LOS experimental environment, the localization error of the ARO-BP model is 6.29 cm, which is 49.48% lower than the 12.45 cm error of the traditional BP model. In the NLOS scenario, the localization error of the ARO-BP algorithmic model is about 10.86 cm, while the localization error of the traditional BP model is about 18.59 cm, and the ARO-BP algorithmic model improves the optimization rate by 46.96% compared to the traditional BP model. The data for each environment and altitude are shown in Table 2.

The empirical findings corroborate the precision and efficacy of the advanced ARO-BP algorithmic framework within the context of ultra-wideband (UWB) indoor positioning technology. However, the current experiments are limited to 5 m × 5 m scenarios, and their robustness in large-scale spaces (e.g., 20 m × 20 m) is yet to be verified. Furthermore, multipath interference suppression in crowded environments still needs to be optimized. In the future, the algorithm will be validated in a 20 m × 20 m warehouse environment, and the real scene will be simulated by deploying more base stations (8–10) and dynamic obstacles.

## Figures and Tables

**Table 1:** Comparison of algorithm’s advantages and disadvantages.

Algorithm	Pros	Cons
BP Neural Network	Simple implementation; Fast training.	Prone to local optima; Low robustness in NLOS.
ARO-BP	Global optimization; High NLOS accuracy.	Higher computational cost; Complex parameter tuning.

**Table 2:** Summary of experimental data.

Environment	Height (m)	BP Error (cm)	ARO-BP Error (cm)	Improvement (%)
LOS	1.8	9.98	5.11	48.80%
LOS	1	14.91	7.48	49.80%
NLOS	0.45	20.81	12.78	38.60%
NLOS	0.15	16.38	9.01	45.00%

## Data Availability

The datasets employed and evaluated in this study can be provided by the corresponding author upon reasonable request.

## Abbreviations

ARO	Artificial Rabbit Optimization
BP	Backpropagation Neural Network
LOS	Line-of-Sight
NLOS	Non-Line-of-Sight
UWB	Ultra-Wideband

## References

[1] Ashraf I., Hur S., Park Y. (2022). Recent Advancements in Indoor Positioning and Localization. Electronics.

[2] Zhao X., Li Q., Wang C., Dou H., Liu B. (2023). Robust Depth-Aided RGBD-Inertial Odometry for Indoor Localization. Measurement.

[3] Ouyang G., Abed-Meraim K., Ouyang Z. (2023). Magnetic-Field-Based Indoor Positioning Using Temporal Convolutional Networks. Sensors.

[4] Perković T., Dujić Rodić L., Šabić J., Solic P. (2023). Machine Learning Approach towards LoRaWAN Indoor Localization. Electronics.

[5] Cheng F., Niu G., Zhang Z., Hou C. (2022). Improved CNN-Based Indoor Localization by Using RGB Images and DBSCAN Algorithm. Sensors.

[6] Sesyuk A., Ioannou S., Raspopoulos M. (2022). A Survey of 3D Indoor Localization Systems and Technologies. Sensors.

[7] Sen U., Yesilirmak Y.E., Bayman I.O., Arsan T., Panayirci E., Stevens N. (2023). 3D indoor positioning with spatial modulation for visible light communications. Opt. Commun..

[8] Feng X., Nguyen K.A., Luo Z. (2022). WiFi Access Points Line-of-Sight Detection for Indoor Positioning Using the Signal Round Trip Time. Remote Sens..

[9] Yoon J., Kim S. (2022). Practical and Accurate Indoor Localization System Using Deep Learning. Sensors.

[10] Jang B., Kim H.-J., Kim J.W. (2023). Survey of Landmark-based Indoor Positioning Technologies. Inf. Fusion.

[11] Gidey H.T., Guo X., Li L., Zhang Y. (2022). Heterogeneous Transfer Learning for Wi-Fi Indoor Positioning Based Hybrid Feature Selection. Sensors.

[12] Yu M., Yao S., Wu X., Chen L. (2022). Research on a Wi-Fi RSSI Calibration Algorithm Based on WOA-BPNN for Indoor Positioning. Appl. Sci..

[13] Hyeon J.J., Kim S. (2018). Indoor Smartphone Localization Based on LOS and NLOS Identification. Sensors.

[14] Cao H., Wang Y., Bi J., Xu S., Si M., Qi H. (2020). Indoor Positioning Method Using WiFi RTT Based on LOS Identification and Range Calibration. ISPRS Int. J. Geo-Inf..

[15] Li X., Bi D., Peng L., Xie Y. (2023). Kernel-based online prediction algorithms for indoor localization in Internet of Things. Expert Syst. Appl..

[16] Yang C., Cheng Z., Jia X., Zhang L., Li L., Zhao Q. (2023). A Novel Deep Learning Approach to 5G CSI/Geomagnetism/VIO Fused Indoor Localization. Sensors.

[17] Suns Q., He L., Meng F., Tong H., Xiao N., Zheng Y. (2022). Wireless communication indoor positioning method in 5G sub-station using deep neural network and location fingerprint algorithm. Optik.

[18] Si M., Wang Y., Xu S., Sun M., Cao H. (2020). A Wi-Fi FTM-Based Indoor Positioning Method with LOS/NLOS Identification. Appl. Sci..

[19] Abudalfa S., Bouchard K. (2023). Two-stage RFID approach for localizing objects in smart homes based on gradient boosted decision trees with under- and over-sampling. J. Reliab. Intell. Environ..

[20] Castillo-Cara M., Talla-Chumpitaz R., Orozco-Barbosa L., García-Castro R. (2023). A novel deep learning approach using blurring image techniques for Bluetooth-based indoor localisation. Inf. Fusion.

[21] Zhen J., Liu B., Wang Y., Liu Y. (2020). An improved method for indoor positioning based on ZigBee technique. Int. J. Embed. Syst..

[22] Qiu K., Huang H., El-Rabbany A. (2020). Geomagnetic Field-Based Indoor Positioning Using Back-Propagation Neural Networks. Int. Arch. Photogramm. Remote Sens. Spat. Inf. Sci..

[23] Zou D., Niu S., Chen S., Su B., Cheng X., Liu J., Liu Y., Li Y. (2019). A smart city used low-latency seamless positioning system based on inverse global navigation satellite system technology. Int. J. Distrib. Sens. Netw..

[24] Morar A., Modoveanu A., Mocanu I.G., Moldoveanu F., Radoi I.E., Asavei V., Gradinaru A., Butean A. (2020). A Comprehensive Survey of Indoor Localization Methods Based on Computer Vision. Sensors.

[25] Ahmed M., Abdalmajeed M., Mahmoud M., Abd El-Rahman A., El-Fikky A., Heba A., Fayed A., Moustafa H.A. (2023). Improved indoor visible light positioning system using machine learning. Opt. Quantum Electron..

[26] Perez-Navarro A., Montoliu R., Torres-Sospedra J. (2022). Advances in Indoor Positioning and Indoor Navigation. Sensors.

[27] Xue J., Tong Z., Kang Z. (2022). A novel indoor positioning algorithm based on UWB. Int. J. Sens. Netw..

[28] Zhang Y., Chu Y., Fu Y., Li Z., Song Y. (2022). UWB Positioning Analysis and Algorithm Research. Procedia Comput. Sci..

[29] Yang J., Yan M. (2018). Implementation of UWB indoor location and distance measurement based on TOF algorithm. MATEC Web Conf..

[30] Zhang F., Yang L., Liu Y., Ding Y., Yang S.-H., Li H. (2022). Design and Implementation of Real-Time Localization System (RTLS) Based on UWB and TDoA Algorithm. Sensors.

[31] Margiani T., Silvano Cortesi M.R.K., Vogt C., Polonelli T., Magno M. (2023). Angle of Arrival and Centimeter Distance Estimation on a Smart UWB Sensor Node. IEEE Trans. Instrum. Meas..

[32] Ibnatta Y., Khaldoun M., Sadik M. (2023). The Indoor Localization System Based on Federated Learning and RSS Using UWB-OFDM. International Conference on Advanced Intelligent Systems for Sustainable Development.

[33] Sandra M.D.I., Stojanovic Z., Jovanovic M., Goran T. (2022). Djordjevic. Multi-algorithm UWB-based localization method for mixed LOS/NLOS environments. Comput. Commun..

[34] Retscher G., Kiss D., Gabela J. (2023). Fusion of GNSS Pseudoranges with UWB Ranges Based on Clustering and Weighted Least Squares. Sensors.

[35] Li B., Hao Z., Dang X. (2019). An indoor location algorithm based on Kalman filter fusion of ultra-wide band and inertial measurement unit. AIP Adv..

[36] Yang B., Li J., Zhang H. (2021). Resilient Indoor Localization System Based on UWB and Visual–Inertial Sensors for Complex Environments. IEEE Trans. Instrum. Meas..

[37] Zhang P., Hu D.Y., Hu P. (2022). Research on UWB fusion location algorithm. J. Phys..

[38] Zhang X., Zheng W.Y., Chen Y. (2022). A Group Learning based Optimization Algorithm Applied to UWB Positioning. J. Phys. Conf. Ser..

[39] Zhang H.H., Wang Q., Yan C., Xu J., Zhang B. (2022). Research on UWB Indoor Positioning Algorithm under the Influence of Human Occlusion and Spatial NLOS. Remote Sens..

[40] Li J., Gao T., Wang X., Guo W., Bai D. (2022). Study on the UWB location algorithm in the NLOS environment. J. Phys..

[41] Du X., Liu M., Sun Y. (2022). Cell Recognition Using BP Neural Network Edge Computing. Contrast Media Mol. Imaging.

[42] Zhang X., Jiang S. (2022). Study on the application of BP neural network optimized based on various optimization algorithms in storm surge prediction. Proc. Inst. Mech. Eng. Part M J. Eng. Marit. Environ..

[43] Zhong L., Wang Y. (2022). Short-term Power Load Forecasting Based on Improved BP Neural Network from Genetic Algorithm and Simulated Annealing Algorithm. J. Phys. Conf. Ser..

[44] Wang W., Zhu Q., Wang Z., Zhao X., Yang Y. (2022). Research on Indoor Positioning Algorithm Based on SAGA-BP Neural Network. IEEE Sens. J..

[45] Ragab M., Abdushkour A.H., Maghrabi L., Alsalman D., Ayman G.F., AL-Ghamdi A.A.-M. (2023). Improved Artificial Rabbits Optimization with Ensemble Learning-Based Traffic Flow Monitoring on Intelligent Transportation System. Sustainability.

[46] Wang L., Cao Q., Zhang Z., Mirjalili S., Zhao W. (2022). Artificial rabbits optimization: A new bio-inspired meta-heuristic algorithm for solving engineering optimization problems. Eng. Appl. Artif. Intell..

[47] Bennet G., Deepa S.N. (2024). Solar PV system with modified artificial rabbit optimizati on algorithm for MPPT. Electr. Eng..

[48] Huang H., Wu R., Huang H., Wei J., Han Z., Wen L., Yuan Y. (2024). Multi-strategy improved artificial rabbit optimization algorithm based on fusion centroid and elite guidance mechanisms. Comput. Methods Appl. Mech. Eng..

